# Supplementary material for: Evolution of longitudinal division in multicellular bacteria of the Neisseriaceae family
Source: Nat Commun. 2022 Aug 22;13:4853. doi: 10.1038/s41467-022-32260-w (PMC9395523; doi:10.1038/s41467-022-32260-w)
Supplement: Supplementary file 1 — Supplementary Information [file 41467_2022_32260_MOESM1_ESM.pdf]

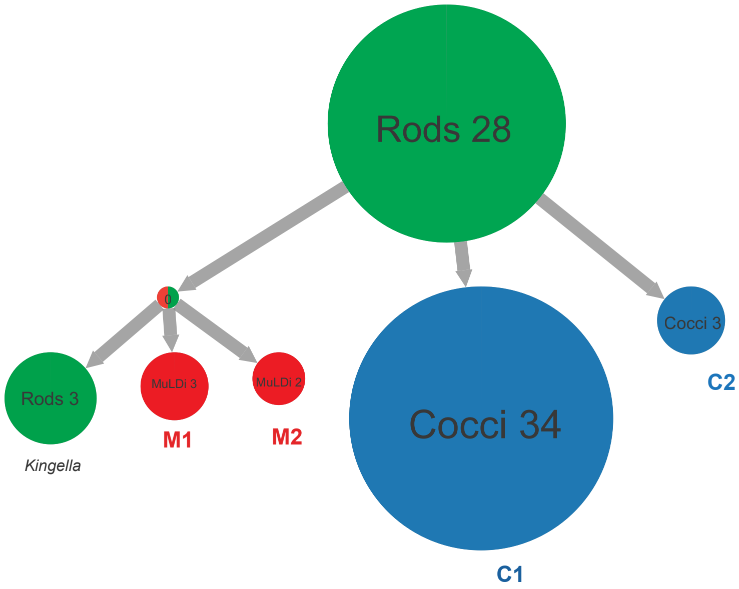

Supplementary Figure 1. The ancestor of the *Neisseriaceae* was rod-shaped. (a) Maximum Likelihood method (PastML; Ishikawa, S. A., Zhukova, A., Iwasaki, W., Gascuel, O. & Pupko, T. (2019). A Fast Likelihood Method to Reconstruct and Visualize Ancestral Scenarios. Molecular Biology and Evolution, 36(9), 2069–2085. https://doi.org/10.1093/molbev/msz131) indicating that the ancestor of all *Neisseriaceae* was rod-shaped.

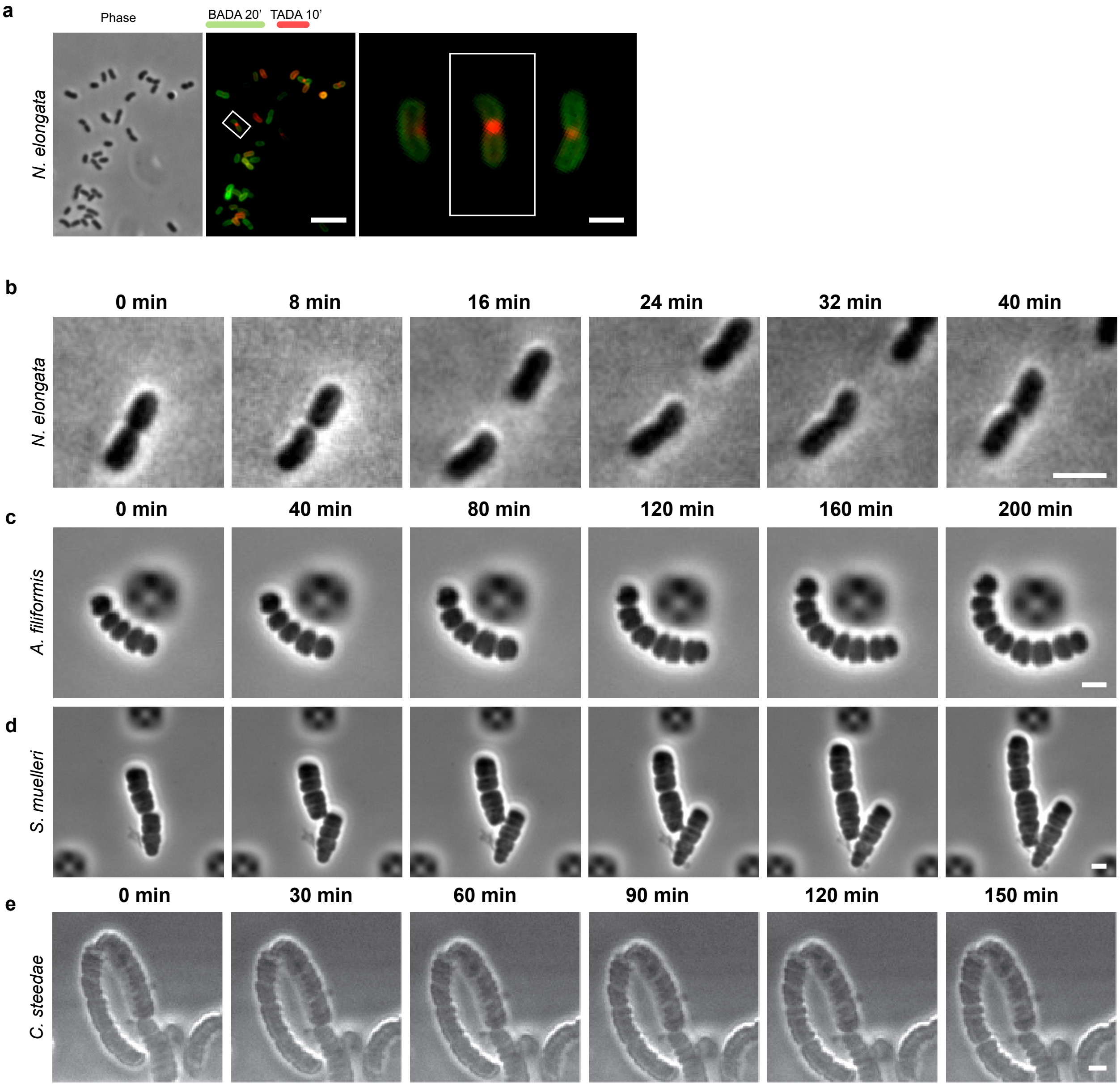

Supplementary Figure 2. Different growth modes of four oral cavity symbionts. (a) *N. elongata* was incubated for 20 min with BADA (green) and, subsequently, for 10 min in TADA (red). Scale bars are 5 µm (middle panel) and 1 µm (right panel). (b-e) Time-lapse microscopy montage showing dividing *N. elongata* (b), *A. filiformis* (c), *S. muelleri* (d) and *C. steedae* (e). Frames show images taken every 8 min for *N. elongata*, every 40 min for *A. filiformis* and *S. muelleri*, and every 30 min for *C. steedae*. The results are representative of at least three independent analyses. See also Supplementary Movies S1, S2, S3 and S4.

a

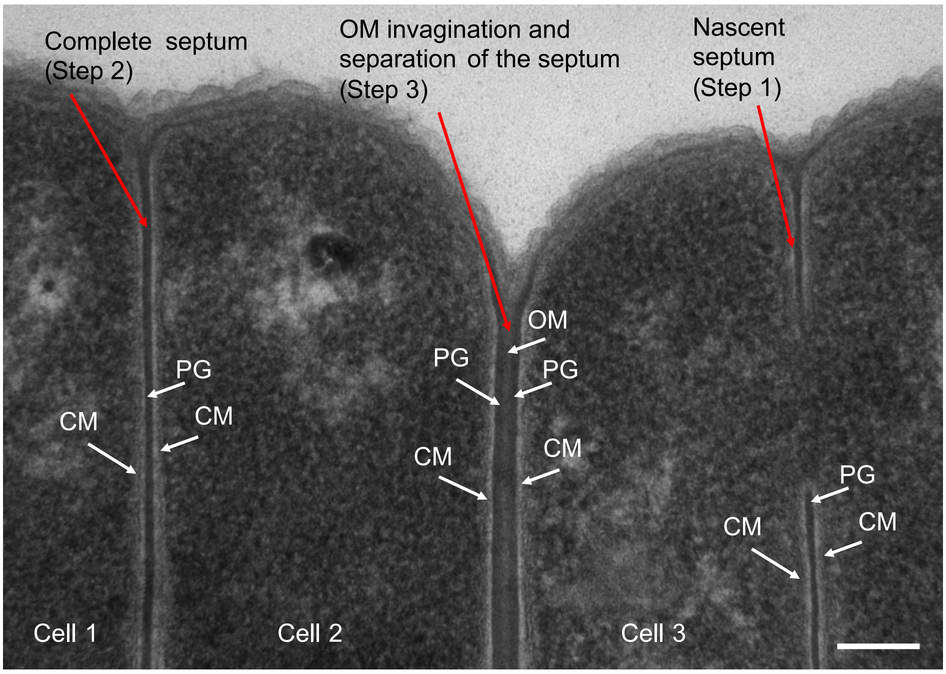

b

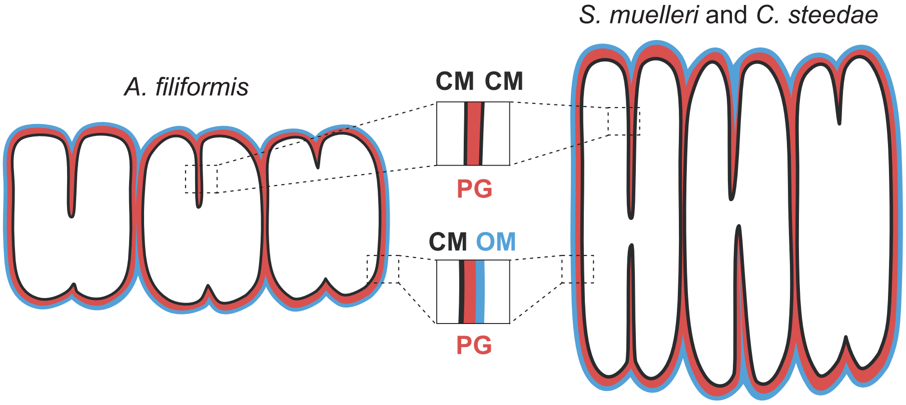

Supplementary Figure 3. Cytoplasmic membrane invagination appears to precede outer membrane invagination in MuLDi *Neisseriaceae*. (a) High magnification transmission electron microscopy image of *S. muelleri*. The results are representative of at least three independent analyses. Scale bar is 100 nm. (b) Schematic representation of the MuLDi envelope.

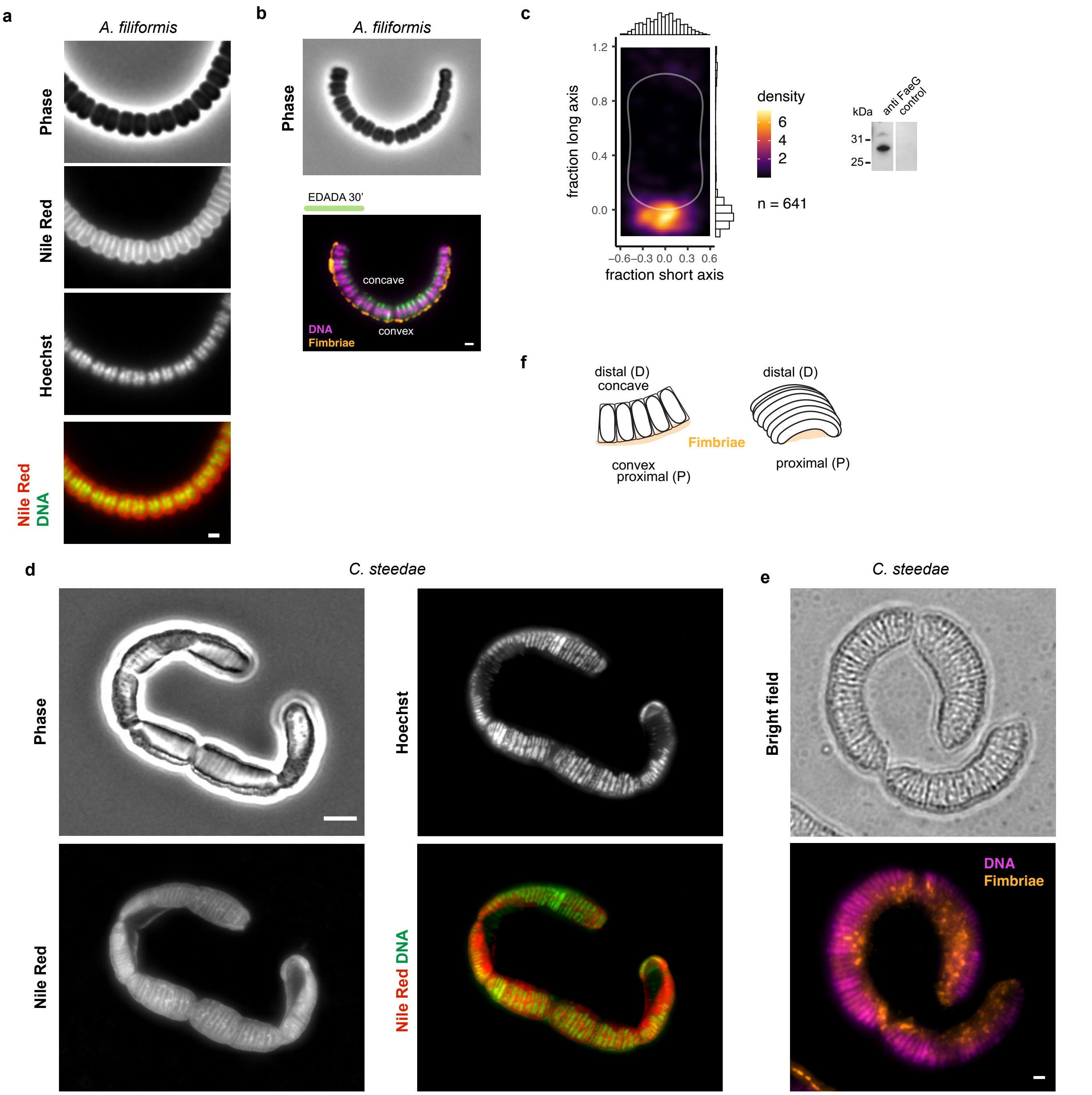

Supplementary Figure 4. Membrane staining and cell polarization of *A. filiformis* and *C. steedae*. (a) Phase contrast image of an *A. filiformis* filament and corresponding membrane staining (Nile red), DNA (Hoechst) staining and overlay are displayed from top to bottom. (b) Phase contrast image (top) and corresponding epifluorescence image (bottom) of *A. filiformis* filament labelled for 30 min with EDA-DA, immunolabelled with an anti-fimbriae antibody and stained with Hoechst. Scale bars are 1 μm. (c) Quantitative analysis of the position of anti-fimbriae antibody fluorescence maxima within 641 individual cells and Western blot of *A. filiformis* protein extracts probed with an anti-fimbriae antibody. (d) Phase contrast image of a *C. steedae* filament (top left) and corresponding membrane (Nile red) staining (bottom left), DNA (Hoechst) staining (top right) and overlay (bottom right). Scale bars are 2 μm (a-d), 1 μm (e) and 5 μm (f). (e) Bright field image (left) and corresponding epifluorescence image (right) of a *C. steedae* filament stained with Hoechst and immunolabelled with an anti-fimbriae antibody. (f) Schematic representations of an *A. filiformis* filament (left) and of a *C. steedae* filament (right). The results are representative of at least three independent analyses. Scale bars are 2 μm (d) and 1 μm (e).

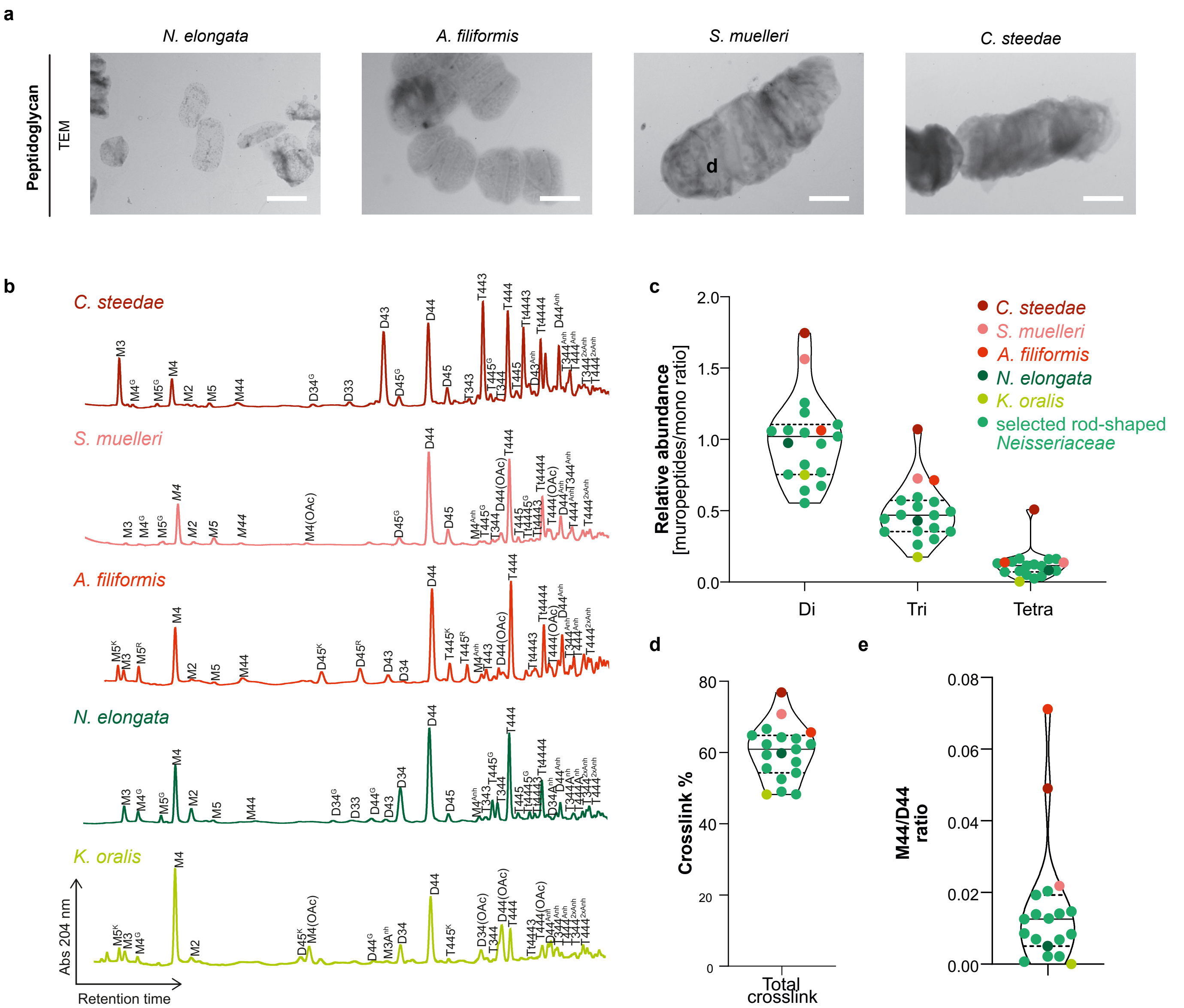

Supplementary Figure 5. TEM of extracted PG of *N. elongata, A. filiformis, S. muelleri* and *C. steedae* and muropeptide analysis of members of the family *Neisseriaceae*. (a) Representative TEM images of sacculi of *N. elongata*, *A. filiformis*, *S. muelleri* and *C. steedae* (from left to right). (b-e) Muropeptide analysis of members of the family *Neisseriaceae*. (b) HPLC chromatograms of muropeptides from *C. steedae* DSM 2580, *S. muelleri* ATCC 29453, *A. filiformis* DSM 16848, *N. elongata* subspglycolytica ATCC 29315, *K. oralis* DSM 18271. Muropeptides characterized by MS are labelled. (c) Distribution of the abundance of dimers (Di), trimers (Tri) and tetramers (Tetra) relative to the abundance of monomers. (d) Overall cross-linking. (e) Relative abundance of the amidase-derived muropeptide product disaccharide octapeptide (M44) relative to its D44 dimer substrate. *C. steedae* DSM 2580, *S. muelleri* ATCC 29453, *A. filiformis* DSM 16848, *N. elongata* subspglycolytica ATCC 29315, *K. oralis* DSM 18271 are labelled in dark red, salmon, red, dark green and yellow green, respectively. Other rod-shaped *Neisseriaceae* (*Neisseria bacilliformis* ATCC BAA-1200, *Neisseria potus* NCTC 13336, *Neisseria musculi*, *Neisseria dentiae* DSMZ 19151, *Neisseria dumasiana* DSMZ 10467, *Neisseria zoodegmatis* DSMZ 21643, *Neisseria species* Dent CA1/247, *Neisseria animaloris* DSMZ 21642, *Neisseria zalophi* DSMZ 102031, *Neisseria weaveri* DSMZ 17688, *Neisseria arctica* DSMZ 103136, *Uruburuella suis* DMSZ 17474, *Uruburuella testudinis* DSMZ 26510, *Neisseria shayeganii* DSMZ 22244 are pale green. The results are representative of at least three independent analyses. Source data are provided as a Source Data file.

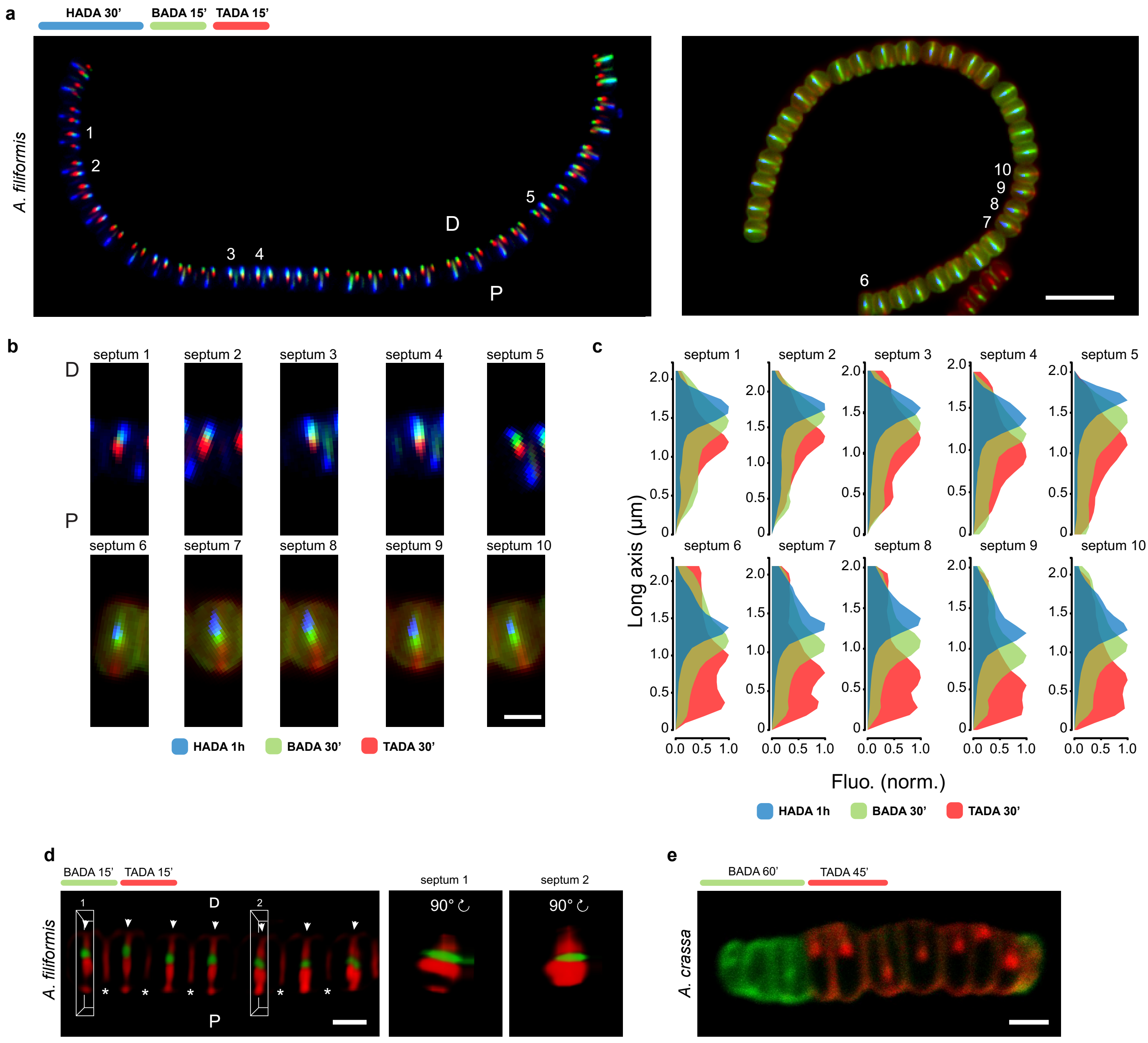

Supplementary Figure 6. Localization of newly synthesized PG in ten *A. filiformis* and in *A. crassa*. (a-c) Epifluorescence microscope-based images of *A. filiformis* consecutively labelled with HADA, BADA and TADA for 30 min, 15 min and 15 min, respectively. (a) Two representative filaments of *A. filiformis*, (b) ten representative *A. filiformis* cells and (c) corresponding septal fluorescence profiles of HADA, BADA and TADA plotted along the long axis. (d) Confocal microscope-based images of *A. filiformis* consecutively labelled with BADA and TADA for 15 min each. Arrowheads point to almost or just completed septa. Asterisks indicate previously competed septa. Fluorescence emitted by an almost completed septum (septum 1; in white box in left panel) and by a just completed septum (septum 2; in white box in left panel) were rotated by 90° and are displayed in the middle and the right panels, respectively. (e) Confocal microscope image of a representative filament of *A. crassa* consecutively labelled with BADA and TADA for 60 min and 45 min, respectively. The results are representative of at least three independent analyses. Source data are provided as a Source Data file. Scale bars correspond to 5 µm (a) or 1 µm (b, d and e).

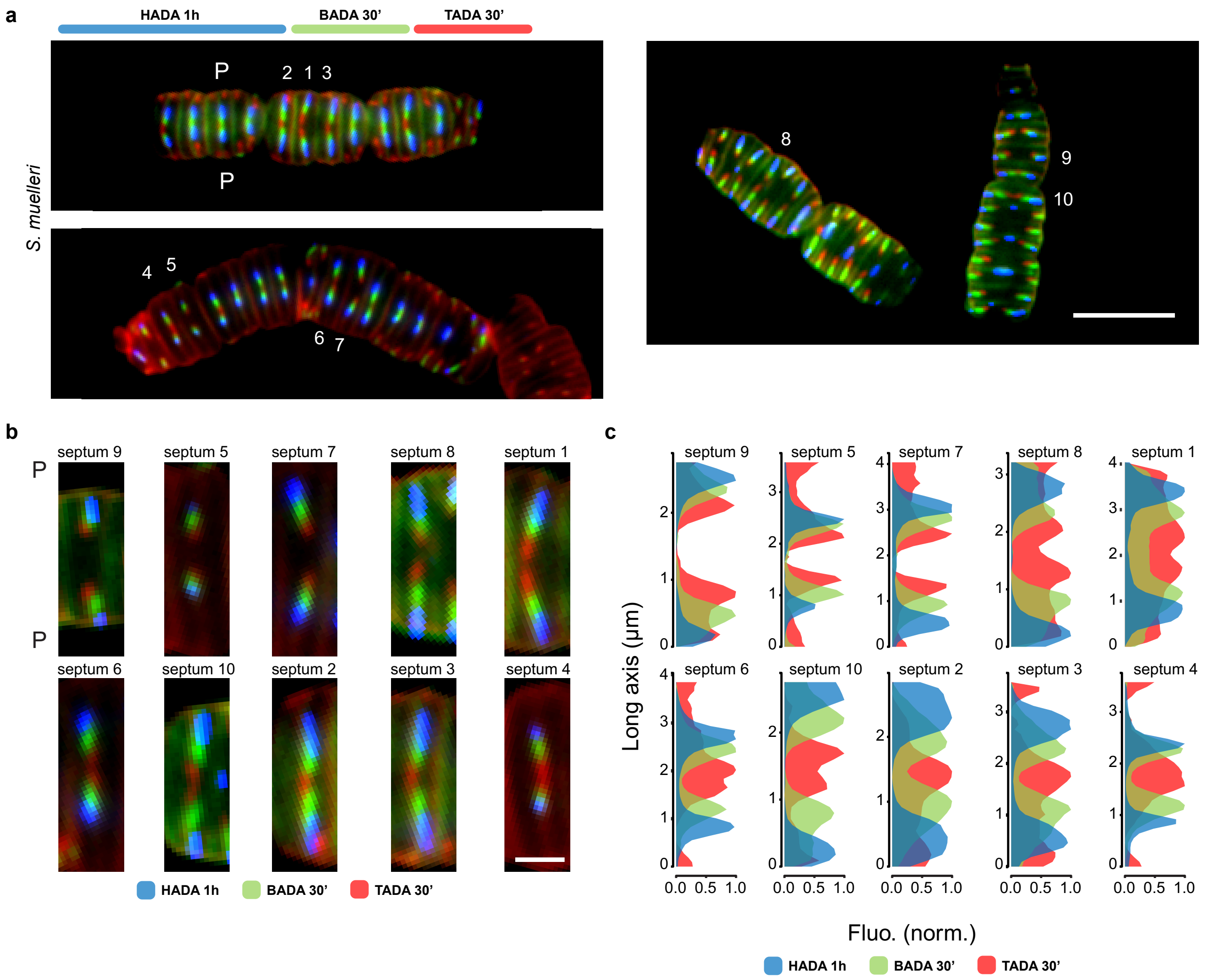

Supplementary Figure 7. Epifluorescence microscope-based localization of newly synthesized PG in ten *S. muelleri*. (a-c) *S. muelleri* was labelled with HADA, BADA and TADA for 1 h, 30 min and 30 min, respectively. (a) Four representative filaments of *S. muelleri*, (b) ten representative cells and (f) corresponding septal fluorescence profiles of HADA, BADA and TADA plotted along the long axis of the 10 cells displayed in (e). The results are representative of at least three independent analyses. Source data are provided as a Source Data file. Scale bars are 5 µm (a) and 1 µm (b).

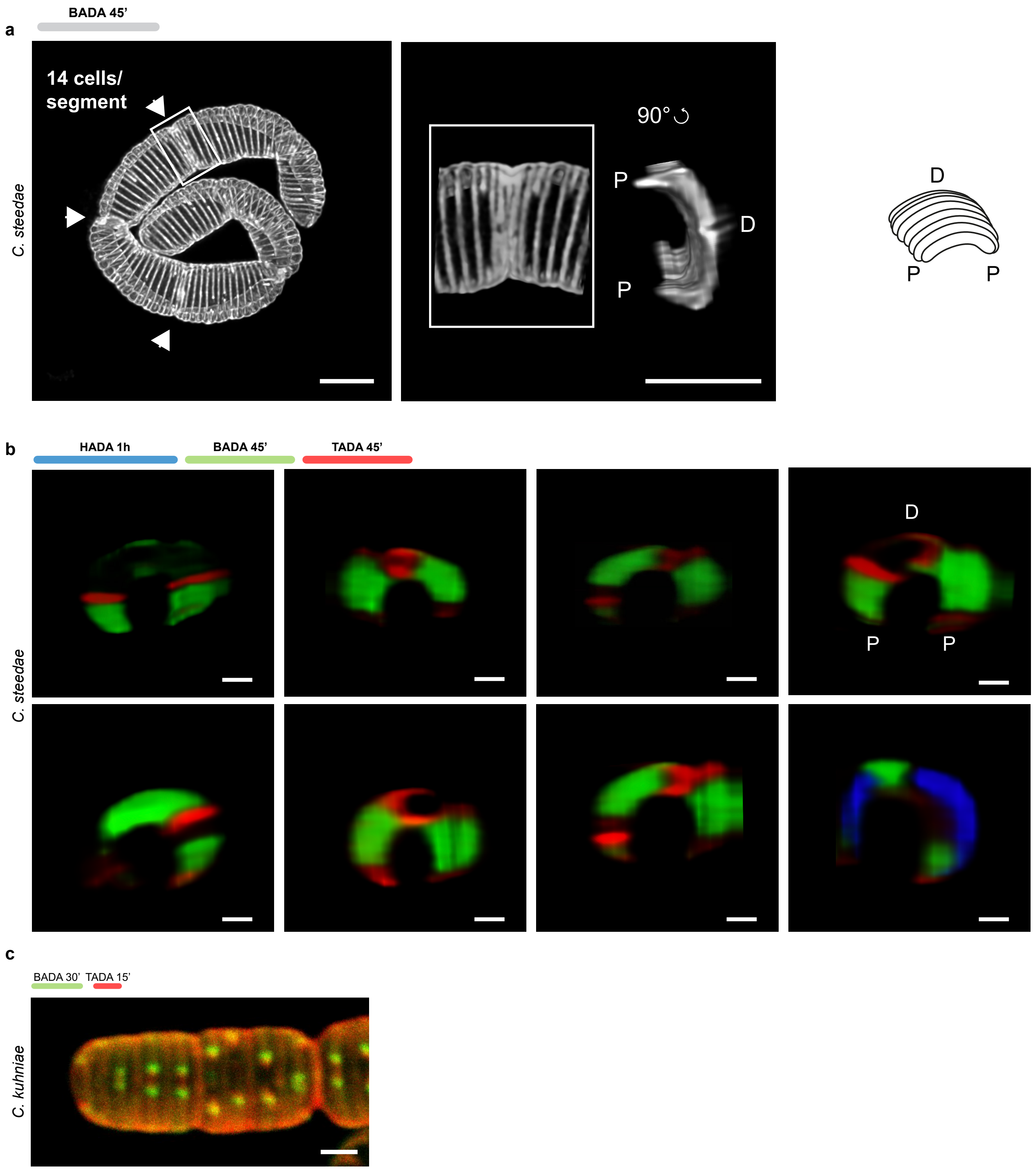

Supplementary Figure 8. Confocal microscopy-based localization of newly synthesized PG in *C. kuhniae* and *C. steedae*. (a) Left panel shows a Z projection of a representative *C. steedae* filament incubated for 45 min with BADA. White arrowheads point at three walls, which appear thicker than the others and which separate two clusters of 14 cells each. Middle and right panels display a lateral view and a 90° rotated view, respectively, of one of the seemingly thicker walls displayed in the left panel (white frame). Scale bar is 5 µm. (b) *C. steedae* septa were cut out of the 3D reconstruction of a filament incubated with HADA for 1h, followed by two pulses with BADA and TADA for 45 minutes. Scale bars represent 1 µm. (c) *C. kuhniae* was labelled consecutively with BADA and TADA for 30 min and 15 min, respectively, and one representative filament was shown. The results are representative of at least three independent analyses. Scale bars correspond to 1 µm.

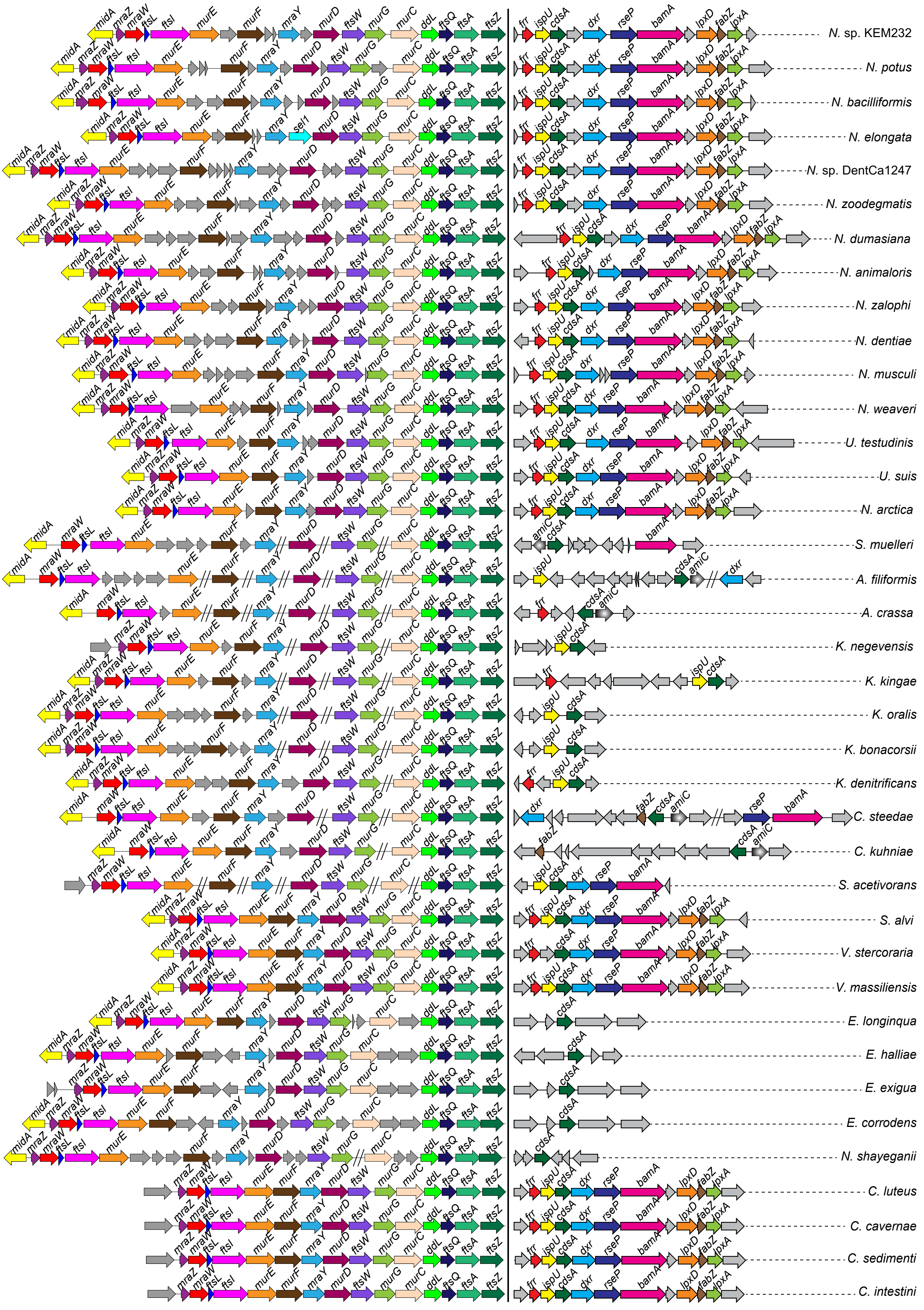

Supplementary Figure 9. The genomic organization of the *dcw* cluster and *cdsA-amiC* loci in the family *Neisseriaceae*.

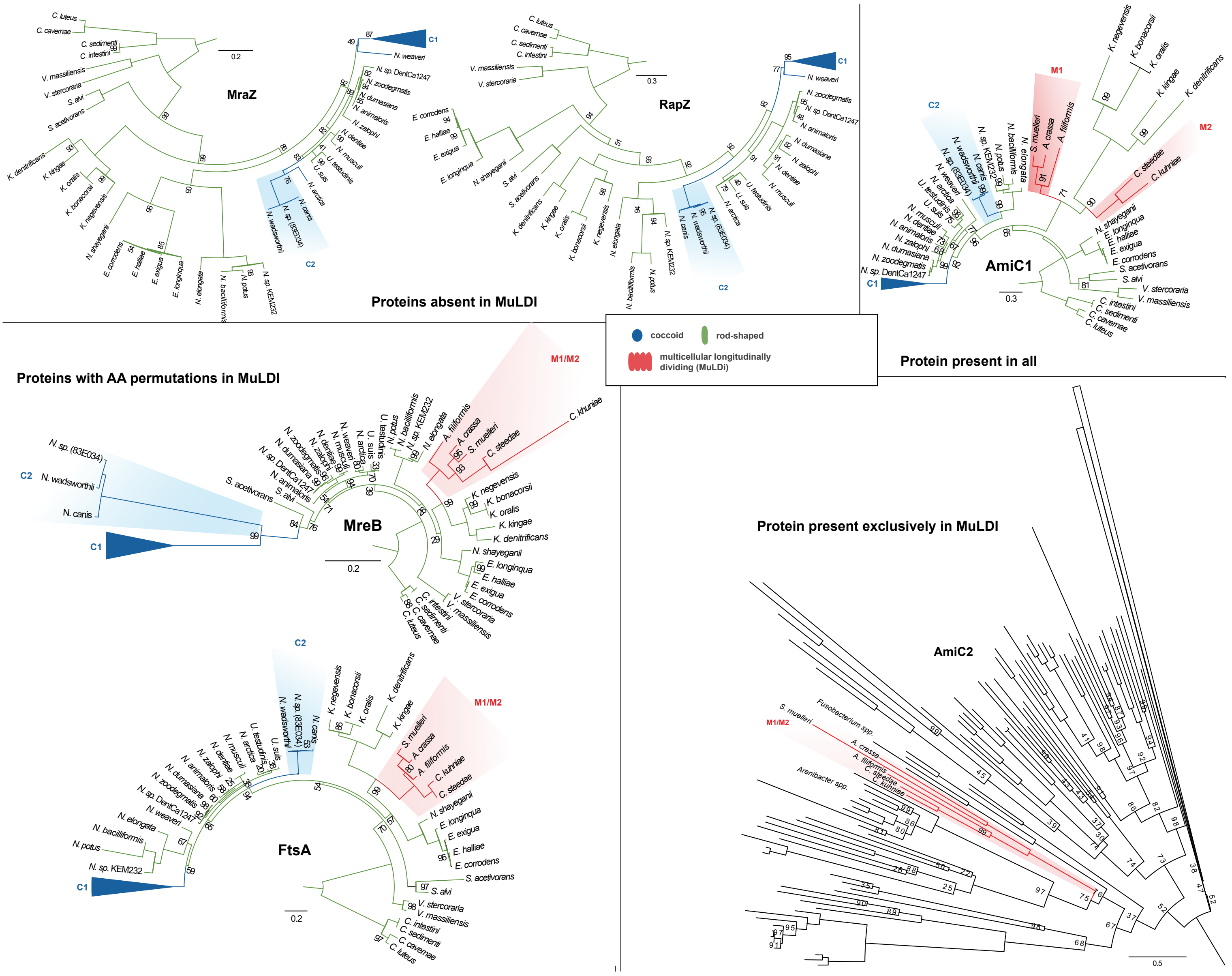

Supplementary Figure 10. RapZ, MraZ, MreB, FtsA, AmiC1 and AmiC2 phylogenies.

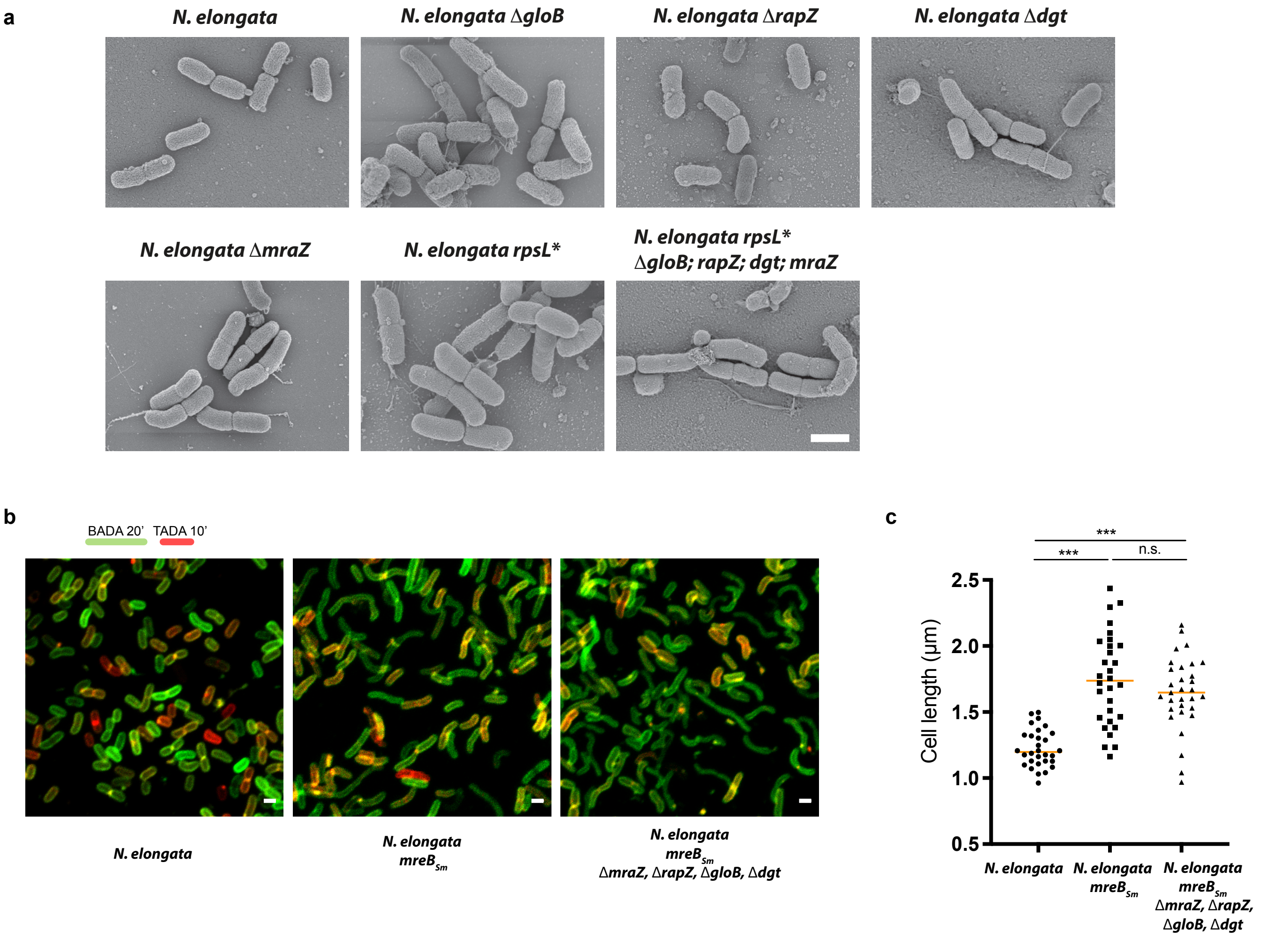

Supplementary Figure 11. Effect of single mutations and of *$mreB_{Ne}$/$mreB_{Sm}$* allelic exchange in wild-type *N. elongata*. (a) Scanning Electron micrographs of *N. elongata* wild-type or harbouring a single deletion in addition to *N. elongata* (*rpsL**) or multiple deletions (Δ*mraZ*, Δ*rapZ*, Δ*gloB*, Δ*dgt*). (b) FDAA labelling of wild-type (*rpsL**) *N. elongata* or harboring the *$mreB_{Ne}$/$mreB_{Sm}$* allelic exchange without (*N. elongata $mreB_{Sm}$*) or with Δ*mraZ*, Δ*rapZ*, Δ*gloB* or Δ*dgt* (*N. elongata* Δ*mraZ*, Δ*rapZ*, Δ*gloB* or Δ*dgt*; *$mreB_{Sm}$*) and (c) cell length measurements (n=30 biologically independent cells. Data are presented with the median). Statistical test used was One-way ANOVA, with Bonferroni's multiple comparisons test (*** p<0.001). The results are representative of at least three independent analyses. Source data and statistics are provided as a Source Data file.

Peptone-Yeast Medium	Peptone from meat	7.5 g
	Yeast extract	1.5 g
	NaCl	2.5 g
	K_2HPO_4	0.5 g
	for prerparing plates add Agar-Agar	6 g
	Distilled water	500 ml
BSTSY Medium (Kuhn et al., 1978)	Tryptone Soja Buillon w/o Dextrose	13.75g
	Yeast extract	2 g
	for prerparing plates add Agar-Agar	7.5 g
	Distilled water	450 ml
	Add 50 mL FBS (Bio-Sell FBS.GP.0100) after autoclaving	
Meat extract Medium	Meat extract (Roth X975.1)	8.5 g
	Yeast extract (Oxoid, Fish.Sc. 10108202)	3.5 g
	NaCl	2.5 g
	K_2HPO_4	1.25 g
	for prerparing plates add Agar-Agar	7.5 g
	Destilled water	500 ml

Supplementary Table 1. Composition of PY, meat extract and BSTSY media

	Label 1	Label 2	Label 3
N. elongata	BADA, 20 min, 1 mM	TADA, 10 min, 1 mM	-
A. filiformis	HADA, 30 min, 2 mM	BADA, 15 min, 1 mM	TADA, 15 min, 1 mM
S. muelleri	HADA, 60 min, 2 mM	BADA, 30 min, 1mM	TADA, 30 min, 1 mM
C. steedae	HADA, 60 min, 2 mM	BADA, 45 min, 1mM	TADA, 45 min, 1 mM
C. kuhniae	BADA, 60 min, 1 mM	TADA, 45 min, 1 mM	-
A. crassa	BADA, 30 min, 1 mM	TADA, 15 min 1 mM	-

Supplementary Table 2. FDAA incubation interval, color and order.

Purpose	Name	Sequence: 5' – 3'
**Generation of mutant strains**	porBp F	TTCGCTAGCGTGCTGAAGCACCAAGTG
	proBp blunt R	CATGGCTGTATTCCTTTTTTGGTTAAG
	proBp lacZF	CTTAACCAAAAAAGGAATACAGCCATGACCATGATTACGGATTCACTG
	LacZRKm7up	ATTTAGATGTCTAAAAAGCATTCAGACGGCACGCGAAATACGGGCAGACAG
	Km7-Up	GCCGTCTGAATGCTTTTTAGACATCTAAAT
	Km6	CCCAGCGAACCATTTGAGG
	5 MraZF	CGCACCAAATTCGTAAACAATACC
	5 MraZR	GACCATAATAAATACGCCTAAACTCCG
	3 MraZF	AAGTTTCAGCTATGAGCAGTCAGGAATTC
	3 MraZR	CTTCAAGCTCACGGTTGATGAAAATC
	5 MraZKmF	CGGAGTTTAGGCGTATTTATTGCCGTCTGAATGCTTTTTAGACATCTAAATCTAGG
	KmpsimR	CTAATCTAAAATTATCTATATACTTCCCAGCGAACCATTTGAGG
	PdcwSm F	GGGAAGTATGTAGATAATTTTAGATTAG
	PdcwSm R	AGACCATAATATTCAATTGGTTTGGCTGAAAGG
	pdcwMcherryF	CCTTTCAGCCAAACCAATTGAATATTATGGTCTCGAAGGGCGAGGAGG
	Mcherry PciI F	GATACATGTTCTCGAAGGGCGAGGAG
	Mcherry NsiI R	GATATGCATTCACTTGTACAGCTCGTC
	CdsAAmiC2F	CTAAGATCTTTATTTATTTTTTAATCTTCC
	AmiC2F	ATGAGATCTGGTAATAATATTAATGCTGTCAAAATC
	AmiC2R	TGGAGATCTTTTTTGAACGAGCTGATTG
	MreBSimonF	ATGATGGATCCTTAAAAATTTAGTTTAGTAAAATCTG
	MreBSimonR	CATGGTTTGCAATGGTGGTGGAAATTATGGATTATTGATAAAAATTGAGTTGA
***N. elongata dcw* qPCR**	RT-*mraZ* F	ATGCCGAAGTTCTGGAAATG
	RT-*mraZ* R	CAATTCGGATGCCAATTCTT
	RT-*mraW* F	GGTGAAGAGCGGTTTAGTCG
	RT-*mraW* R	GAAAATCCGAATGGCTTGAA
	RT-*ftsL* F	CCGTGGTTACCCAGCAAA
	RT-*ftsL* R	GCTCGGCTGTACCAATTTTC
	RT-*ftsI* F	AAGCCGTCTGAACTGGAAAA
	RT-*ftsI* R	GGGTTTCATCTGCCGTTTTA
	RT-*ftsQ* F	AAATCCGATTGAGTGAGCGC
	RT-*ftsQ* R	TGTCCTTTGAATTGCGGCAA
	RT-*ftsA* F	GGCCGAATTGATGGCTGATT
	RT-*ftsA* R	CGATATCCGCCTGACTGACT
	RT-*ftsZ* F	CGCTGGTGTGATTACGTCTG
	RT-*ftsZ* R	AATGCTTCCTCTTTGACGGC
	RT-*gyrA* Nelong F	GCAACCATCTACGGCTTGAG
	RT-*gyrA* Nelong R	ATGATGATGGCTTCGCGTTC

Supplementary Table 3. Primer sequences used for generating the mutant strains and for *N. elongata dcw* quantitative real-time PCR
