# Supplementary material for: Evolution of longitudinal division in multicellular bacteria of the Neisseriaceae family
Source: Nat Commun. 2022 Aug 22;13:4853. doi: 10.1038/s41467-022-32260-w (PMC9395523; doi:10.1038/s41467-022-32260-w)
Supplement: Supplementary file 2 — Reporting Summary [file 41467_2022_32260_MOESM2_ESM.pdf]

nature portfolio

Corresponding author(s): Silvia Bulgheresi

Last updated by author(s): Apr 27, 2022

Reporting Summary

Nature Portfolio wishes to improve the reproducibility of the work that we publish. This form provides structure for consistency and transparency in reporting. For further information on Nature Portfolio policies, see our Editorial Policies and the Editorial Policy Checklist.

Statistics

For all statistical analyses, confirm that the following items are present in the figure legend, table legend, main text, or Methods section.

n/a	Confirmed	
☐	☒	The exact sample size (n) for each experimental group/condition, given as a discrete number and unit of measurement
☐	☒	A statement on whether measurements were taken from distinct samples or whether the same sample was measured repeatedly
☐	☒	The statistical test(s) used AND whether they are one- or two-sided<br>*Only common tests should be described solely by name; describe more complex techniques in the Methods section.*
☒	☐	A description of all covariates tested
☒	☐	A description of any assumptions or corrections, such as tests of normality and adjustment for multiple comparisons
☐	☒	A full description of the statistical parameters including central tendency (e.g. means) or other basic estimates (e.g. regression coefficient) AND variation (e.g. standard deviation) or associated estimates of uncertainty (e.g. confidence intervals)
☐	☒	For null hypothesis testing, the test statistic (e.g. F, t, r) with confidence intervals, effect sizes, degrees of freedom and P value noted<br>*Give P values as exact values whenever suitable.*
☒	☐	For Bayesian analysis, information on the choice of priors and Markov chain Monte Carlo settings
☒	☐	For hierarchical and complex designs, identification of the appropriate level for tests and full reporting of outcomes
☒	☐	Estimates of effect sizes (e.g. Cohen's d, Pearson's r), indicating how they were calculated

Our web collection on statistics for biologists contains articles on many of the points above.

Software and code

Policy information about availability of computer code

Data collection	To acquire images using fluorescent and electron microscope the following software were used:<br>- NIS Element 5.02.01 software (Nikon)<br>- SEM operation software Regulus 8200 series<br>- TEM - AMT Image Capture Engine version 600.147<br>- Zeiss Software Zen 2011 2.3 SP1<br>- Leica Software LASX version 3.7.2.22383 including the Lightning deconvolution software package<br>- ProgRes Capture Pro 2.8.8 software<br>For HPLC :<br>Waters Empower 3, build 3471 software<br>For MS:<br>UNIFI software platform<br>For DNA sequencing (nanopore device):<br>- MinKNOW 21.05.10<br>- Guppy 5.0.11<br>For RNA sequencing and qRT-PCR:<br>- MinKNOW 21.05.10<br>- Guppy 5.0.11<br>- StepOneTM Software v2.3 (QRT)
Data analysis	To analyse images obtained using fluorescent microscopy the following software were used:<br>- NIS Element Software version 5.02.01 software (Nikon)<br>- Fiji Software package version 1.53q

- ImageJ plugin Fil-Tracer (https://sils.fnwi.uva.nl/bcb/objectj/examples/Fil-Tracer/MD/Fil-Tracer.html)
For Statistics, representation and others:
- Graphpad Prism 9.0/9.3.0/9.4.0
- Excel 2021
- ggplot2 in R (http://www.R-project.org/)
- Adobe Photoshop and Illustrator 2021
- Venny 2.1
For genomics :
- HGAP v.4
- SMRT Link v.7
- guppy_basecaller (version 5.0.11+2b6dbff)
- Flye (https://github.com/fenderglass/Flye)
- Miniasm (https://github.com/lh3/miniasm)
- Pilon (https://github.com/broadinstitute/pilon)
- Racon (https://github.com/isovic/racon)
- Medaka (https://github.com/nanoporetech/medaka)
- Quast (https://github.com/ablab/quast)
- BUSCO (https://gitlab.com/ezlab/busco)
- MycoHIT pipeline (https://www.mcgill.ca/molepi/mycohit-software)
- EMBOSS 6.6.0.0
- EasyFig 2.2.2
For Phylogenetics/phylogenomics :
- Prokka v1.14.5
- Roary v3.11.2
- ModelFinder within IQ-TREE version 1.6.3
- IQ-TREE webserver
- FigTree v1.4.4
For Transcriptomics :
- FastQC (Version 0.73)
- FastQ Groomer (Version 1.1.5)
- Bowtie2 (Version 2.4.2)
- htseq_count (Version 0.9.1)
- DESeq2 (Version 2.22.40.6)
- guppy_basecaller (version 5.0.11+2b6dbff)
- guppy_barcoder (version 5.0.11+2b6dbff)
- Prokka v1.14.5
- NetworkX version 2.6.2
- minimpa2 (https://github.com/lh3/minimap2)
- featureCounts v2.0.1 of Subread package
- DESeq2 version 3.14
- STRING webserver
The codes used in this study have been reported previously and are available as described in the corresponding M&M section. The documentation for the ImageJ plugin Fil-Tracer can be accessed here: https://sils.fnwi.uva.nl/bcb/objectj/examples/Fil-Tracer/MD/Fil-Tracer.html. The other custom codes generated during the current study are available from the corresponding authors on reasonable request.

For manuscripts utilizing custom algorithms or software that are central to the research but not yet described in published literature, software must be made available to editors and reviewers. We strongly encourage code deposition in a community repository (e.g. GitHub). See the Nature Portfolio guidelines for submitting code & software for further information.

Data

Policy information about availability of data

All manuscripts must include a data availability statement. This statement should provide the following information, where applicable:

- Accession codes, unique identifiers, or web links for publicly available datasets
- A description of any restrictions on data availability
- For clinical datasets or third party data, please ensure that the statement adheres to our policy

Data availability. The genome datasets generated during and/or analysed during the current study (see supplementary data 1) are available in the NCBI genome repository (https://www.ncbi.nlm.nih.gov/genome/browse#!/overview/) under the accession codes:
GCA_022870985.1, [https://www.ncbi.nlm.nih.gov/assembly/GCF_022870985.1/]
GCA_014055025.1, [https://www.ncbi.nlm.nih.gov/assembly/GCF_014055025.1/]
GCA_000818035.1, [https://www.ncbi.nlm.nih.gov/assembly/GCF_000818035.1/]
GCA_022870825.1, [https://www.ncbi.nlm.nih.gov/assembly/GCF_022870825.1/]
GCA_022870885.1, [https://www.ncbi.nlm.nih.gov/assembly/GCF_022870885.1/]
GCA_900637855.1, [https://www.ncbi.nlm.nih.gov/assembly/GCF_900637855.1/]
GCA_008807015.1, [https://www.ncbi.nlm.nih.gov/assembly/GCF_008807015.1/]
GCA_014055005.1, [https://www.ncbi.nlm.nih.gov/assembly/GCF_014055005.1/]
GCA_014297595.1, [https://www.ncbi.nlm.nih.gov/assembly/GCF_014297595.1/#/qa]
GCA_001308015.1, [https://www.ncbi.nlm.nih.gov/assembly/GCF_001308015.1/]
GCA_014054885.1, [https://www.ncbi.nlm.nih.gov/assembly/GCF_014054885.1/]

GCA_900636765.1, [https://www.ncbi.nlm.nih.gov/assembly/GCF_900636765.1/]
GCA_900638685.1, [https://www.ncbi.nlm.nih.gov/assembly/GCF_900638685.1/]
GCA_022870865.1, [https://www.ncbi.nlm.nih.gov/assembly/GCF_022870865.1/]
GCA_022870845.1, [https://www.ncbi.nlm.nih.gov/assembly/GCF_022870845.1/]
GCA_022870905.1, [https://www.ncbi.nlm.nih.gov/assembly/GCF_022870905.1/]
GCA_002951835.1, [https://www.ncbi.nlm.nih.gov/assembly/GCF_002951835.1/]
GCA_014054525.1, [https://www.ncbi.nlm.nih.gov/assembly/GCF_014054525.1/]
GCA_022871045.1, [https://www.ncbi.nlm.nih.gov/assembly/GCF_022871045.1/]
GCA_900177895.1, [https://www.ncbi.nlm.nih.gov/assembly/GCF_900177895.1/]
GCA_022871005.1, [https://www.ncbi.nlm.nih.gov/assembly/GCF_022871005.1/]
GCA_014054985.1, [https://www.ncbi.nlm.nih.gov/assembly/GCF_014054985.1/]
GCA_016623605.1, [https://www.ncbi.nlm.nih.gov/assembly/GCF_016623605.1/]
GCA_016127355.1, [https://www.ncbi.nlm.nih.gov/assembly/GCF_016127355.1/]
GCA_014054725.1, [https://www.ncbi.nlm.nih.gov/assembly/GCF_014054725.1/]
GCA_022871025.1, [https://www.ncbi.nlm.nih.gov/assembly/GCA_022871025.1/]
GCA_000745895.1, [https://www.ncbi.nlm.nih.gov/assembly/GCF_000745895.1/]
GCA_022870965.1, [https://www.ncbi.nlm.nih.gov/assembly/GCF_022870965.1/]
GCA_022870945.1, [https://www.ncbi.nlm.nih.gov/assembly/GCF_022870945.1/]
GCA_022870925.1, [https://www.ncbi.nlm.nih.gov/assembly/GCF_022870925.1/]
GCA_001648355.1, [https://www.ncbi.nlm.nih.gov/assembly/GCF_001648355.1/]
GCA_001648475.1, [https://www.ncbi.nlm.nih.gov/assembly/GCF_001648475.1/]
GCA_008805035.1, [https://www.ncbi.nlm.nih.gov/assembly/GCF_008805035.1/]
GCA_900187105.1, [https://www.ncbi.nlm.nih.gov/assembly/GCF_900187105.1/]
GCA_014054965.1, [https://www.ncbi.nlm.nih.gov/assembly/GCF_014054965.1/]
Source data analysed during the current study and the corresponding statistics are provided as a Source Data file. Raw reads data are available on SRA database under the accession codes : PRJNA788950; [https://www.ncbi.nlm.nih.gov/sra/PRJNA788950], PRJNA859696; [https://www.ncbi.nlm.nih.gov/sra/PRJNA859696], PRJNA859916; [https://www.ncbi.nlm.nih.gov/sra/PRJNA859916], PRJNA859935; [https://www.ncbi.nlm.nih.gov/sra/PRJNA859935].

Field-specific reporting

Please select the one below that is the best fit for your research. If you are not sure, read the appropriate sections before making your selection.

- [x] Life sciences
- [ ] Behavioural & social sciences
- [ ] Ecological, evolutionary & environmental sciences

For a reference copy of the document with all sections, see nature.com/documents/nr-reporting-summary-flat.pdf

Life sciences study design

All studies must disclose on these points even when the disclosure is negative.

Sample size

Sample sizes were similar to those generally employed in the field. In the case of imaging experiments requiring statistical analysis, the numbers are at least equal to n=30 (up to n=376). In the case of RT-qPCR and RNAseq comparing mutants, three biological replicas were used for the analysis of the results. All the experiments have been replicated at least twice.

Data exclusions

In this manuscript, we have excluded coccoid lineages of Neisseriaceae and focused on the analysis of the remaining 41 species (Figure 1, Supplementary Data 1). The data obtained when we performed rod versus MuLDi Neisseriaceae comparative genomics while excluding Kingella spp. are presented in Supplementary Data 5.

Replication

All experiments have been replicated at least twice.

Randomization

Randomization was not necessary for the experiments. In the case of genomics, transcriptomics and phylogenetics analyzes randomization is not possible. In the case of image analyses, all the bacteria present on the images, and respecting the previously established criteria, were measured. It was therefore not useful to randomly select cells to be measured.

Blinding

Blinding was not performed as it was not necessary because the experimental conditions were known to the researchers. In the case of genomics and transcriptomics analyses, all the data generated was analyzed with the same workflow. In the case of measurements made on the images, a measurement protocol was established and applied in a similar way to all the images. All the cells respecting the selection criteria established in advance were measured. Data were analyzed using unbiased statistical tests.

Reporting for specific materials, systems and methods

We require information from authors about some types of materials, experimental systems and methods used in many studies. Here, indicate whether each material, system or method listed is relevant to your study. If you are not sure if a list item applies to your research, read the appropriate section before selecting a response.

Materials & experimental systems

n/a	Involved in the study	
☐	☒	Antibodies
☒	☐	Eukaryotic cell lines
☒	☐	Palaeontology and archaeology
☒	☐	Animals and other organisms
☒	☐	Human research participants
☒	☐	Clinical data
☒	☐	Dual use research of concern

Methods

n/a	Involved in the study	
☒	☐	ChIP-seq
☒	☐	Flow cytometry
☒	☐	MRI-based neuroimaging

Antibodies

Antibodies used	Sheep polyclonal anti-E. coli K88 fimbrial protein AB/FaeG antibody (ab35292, Abcam)
Validation	Primary antibody was validated by Western blot.
