# Supplementary material for: Evolution of longitudinal division in multicellular bacteria of the Neisseriaceae family
Source: Nat Commun. 2022 Aug 22;13:4853. doi: 10.1038/s41467-022-32260-w (PMC9395523; doi:10.1038/s41467-022-32260-w)
Supplement: Supplementary file 3 — Description of Additional Supplementary Files [file 41467_2022_32260_MOESM3_ESM.pdf]

**Title:** Supplementary Movie 1.
**Description:** Growth of *N. elongata*. Time-lapse of *N. elongata* cells grown in BSTSY at 37°C with 5% CO2. This movie was used to create Supplementary Figure 2b.

**Title:** Supplementary Movie 2.
**Description:** Growth of *A. filiformis*. Time-lapse of *A. filiformis* cells grown in PY at 37°C. This movie was used to create the panels shown in Supplementary Figure 2c.

**Title:** Supplementary Movie 3.
**Description:** Growth of *S. muelleri*. Time-lapse of S. muelleri cells grown in meat extract at 37°C with 5% CO2. This movie was used to create the panels shown in Supplementary Figure 2d.

**Title:** Supplementary Movie 4.
**Description:** Growth of *C. steedae*. Time-lapse of C. steedae cells grown in BSTSY at 37°C. This movie was used to create the panels shown in Supplementary Figure 2e.

**Title:** Supplementary Movie 5.
**Description:** Confocal microscopy-based 3D reconstruction of an *A. filiformis* filament subjected to triple FDAA labelling. Stills from this movie are shown in Figure 3d.

**Title:** Supplementary Movie 6.
**Description:** Confocal microscopy-based 3D reconstruction of a *C. steedae* filament subjected to triple FDAA labelling. Stills from this movie S6 are shown in Figure 4e.

**Title:** Supplementary Movie 7.
**Description:** Confocal microscopy-based 3D reconstruction of an *C. steedae* filament subjected to FDAA labelling (45 min with BADA). Stills from this movie are shown in Supplementary Figure 8.

**Title:** Supplementary Data 1.
**Description:** Genomes used for comparative genomics.

**Title:** Supplementary Data 2.
**Description:** Average Nucleotide Identity (ANI) between pairs of publicly available *Neisseriaceae* draft genomes.

**Title:** Supplementary Data 3.
**Description:** Genes used for the *Neisseriaceae* phylogeny.

**Title:** Supplementary Data 4.
**Description:** Table of identified muropeptides. Identity was confirmed by MS analysis. Short name, structure, theoretical, observed neutral mass in Da and retention time are indicated.

**Title:** Supplementary Data 5.
**Description:** Genes present or absent in MuLDi *Neisseriaceae* when *Kingella* spp. are excluded from the comparative genomic analysis presented in Figure 5a.

**Title:** Supplementary Data 6:
**Description:** Phylogenies raw data.
